# Socioeconomic inequalities in early initiation and exclusive breastfeeding practices in Bangladesh: findings from the 2018 demographic and health survey

**DOI:** 10.1186/s13006-021-00420-1

**Published:** 2021-09-26

**Authors:** Michael Ekholuenetale, Sabuj Kanti Mistry, Ritesh Chimoriya, Simone Nash, Ashish M. Doyizode, Amit Arora

**Affiliations:** 1 Department of Epidemiology and Medical Statistics, Faculty of Public Health, College of Medicine, University of Ibadan, Ibadan, Nigeria; 2 Centre for Primary Health Care and Equity, University of New South Wales, Sydney, Australia; 3 BRAC James P Grant School of Public Health, BRAC University, 68 Shahid Tajuddin Ahmed Sharani, Mohakhali, Dhaka, 1212 Bangladesh; 4 School of Medicine, Western Sydney University, Campbelltown, NSW 2560 Australia; 5 Health Equity Laboratory, School of Health Sciences, Western Sydney University, Locked Bag 1797, Penrith, NSW 2751 Australia; 6 Translational Health Research Institute, Western Sydney University, Locked Bag 1797, Penrith, NSW 2751 Australia; 7 Discipline of Child and Adolescent Health, Sydney Medical School, Faculty of Medicine and Health, Westmead, NSW 2145 Australia; 8 Oral Health Services, Sydney Local Health District and Sydney Dental Hospital, NSW Health, Surry Hills, NSW 2010 Australia

**Keywords:** Early initiation of breastfeeding, Exclusive breastfeeding, Breastfeeding, Early childhood, Socioeconomic inequalities, Sustainable development goals, Women, Bangladesh

## Abstract

**Background:**

Optimal breastfeeding practices including early initiation of breastfeeding and exclusive breastfeeding (EBF) are associated with positive health outcomes. Socioeconomic inequalities in key breastfeeding indicators may play a role in the prevalence of breastfeeding practices. The objective of this study was to examine the socioeconomic inequalities in early initiation of breastfeeding and EBF practices in Bangladesh based on the 2018 Bangladesh Demographic and Health Survey (BDHS).

**Methods:**

This was a secondary data analysis of the nationally representative 2018 BDHS. Data on 4950 women of reproductive age who had ever given birth and 924 children aged 0–5 months were extracted, for early initiation of breastfeeding and EBF. Early initiation of breastfeeding was determined from children who were put to the breast within the first hour of birth. Exclusive breastfeeding was estimated from children aged 0–5 months who were exclusively breastfed.

**Results:**

The weighted prevalence of early initiation of breastfeeding and EBF were 60.8% (95% CI; 59.0, 62.6%) and 66.8% (95% CI; 63.1, 70.3%), respectively. The estimated prevalence of early initiation among the poorest, poorer, middle, richer and richest households were 67.8, 66.3, 58.4, 56.3 and 54.4%, respectively. Similarly, early initiation prevalence of 64.4, 65.0, 61.1 and 52.3% were estimated among women with no formal education, primary, secondary and higher education, respectively. The estimated prevalence of EBF among the poorest, poorer, middle, richer and richest households were 63.0, 65.2, 67.7, 66.7 and 69.9%, respectively. Similarly, the estimated EBF prevalence were 62.5, 66.0, 66.3 and 68.9% among women with no formal education, primary, secondary and higher education, respectively. Early initiation of breastfeeding was higher among lower household wealth (Conc. Index = − 0.049; SE = 0.006) and lower educational attainment groups (Conc. Index = − 0.035; SE = 0.006).

**Conclusions:**

Improving optimal breastfeeding practices in Bangladesh should be given utmost priority. A need to address the socioeconomic inequalities in breastfeeding practices was also identified.

## Background

The importance of optimal breastfeeding practices for both maternal and child health have long been recognised [1]. Breast milk contains all essential nutrients required for the proper growth and development of infants and young children [2], and the presence of immunoglobulin and anti-inflammatory properties protect both children and mothers from infections and diseases [3]. The World Health Organization (WHO) recommends early initiation of breastfeeding as soon as possible after birth, within the first hour after delivery [4]. Colostrum, produced in low quantities during the first few days of birth, is rich in nutrients and antibodies [5]. Evidence-based research has linked early initiation of breastfeeding with reduced risk of morbidity and mortality among children [6]. Moreover, it may also help reduce the maternal mortality ratio, as it is evident that early initiation of breastfeeding can prevent postpartum haemorrhage which is a prime cause of maternal mortality [7]. Similarly, the WHO recommends that infants be exclusively breastfed for the first six months of their life [8]. Exclusive breastfeeding (EBF) is defined as the practice where an infant receives only breast milk for the first six months, with no other liquids (not even water) or solid foods, except oral rehydration solutions, drops, and syrups (vitamins, minerals, and medicines) [4]. Exclusively breastfed children are at lower risk of several health conditions [9], and it is estimated that the benefits of EBF could help avert 13–15% of deaths among children in low- and middle-income countries (LMICs) [10].

There is overwhelming evidence clearly demonstrating the importance of early initiation of breastfeeding and EBF for child growth and survival. However, only around 40% of infants globally receive breast milk within the first hour of birth [11]. Globally, approximately 50% of infants up to one month of age and only 30% of infants aged 1–5 months are exclusively breastfed [12]. Similar to other LMICs [13–16], there is a low prevalence of early initiation of breastfeeding and exclusive breastfeeding in Bangladesh [3, 5, 17, 18]. Recent studies in Bangladesh have revealed that the prevalence of early initiation of breastfeeding was 51.24% [19] and EBF was 61% [17]. In light of these findings, continuous monitoring of breastfeeding rates to determine to what extent breastfeeding recommendations are being met, and further investigating if any socioeconomic inequalities or disparities in breastfeeding practices exist are essential measures to improve the uptake and practice of early initiation of breastfeeding and EBF in Bangladesh.

Several studies conducted in Bangladesh have investigated the socioeconomic determinants of early initiation of breastfeeding [5, 20]. A study based on the 2014 BDHS [5] found that mothers from rural areas in Bangladesh were more likely to practice early initiation of breastfeeding. The authors also indicated that early initiation of breastfeeding practices were relatively higher among the less educated women, and this was linked to the finding that early initiation of breastfeeding practices were higher among women from rural areas who were less educated compared to those of urban areas [5]. On the other hand, the 2014 BDHS [20] found that exposure to mass media was associated with improvement in early initiation of breastfeeding, and the authors highlighted the importance of mobile phone and mass media campaigns in disseminating feeding-related information to improve early initiation of breastfeeding practices. Significant geographical variation has also been reported in terms of early initiation of breastfeeding practices in Bangladesh [5]. Early initiation of breastfeeding rates were significantly higher among mothers residing in Sylhet division, which could be attributed to the local culture and norms as well as the lower rates of caesarean delivery in this part of Bangladesh.

Similarly, a number of socioeconomic factors have been associated with EBF practices in Bangladesh. Several studies found that EBF practices were significantly lower among women with higher education and who were employed in formal jobs [3, 17]. Similar findings were also reported in other LMICs and South Asian countries [21, 22]. Mothers’ age was also identified as a significant determinant of EBF practice in Bangladesh, and significantly higher rates were found among older mothers [3]. Similar to that of early initiation of breastfeeding practice, EBF practices were also relatively higher among mothers from Sylhet division and those who were exposed to mass media. However, a recent study carried out in rural areas of Rajshahi district reported that EBF practices were significantly higher among women engaged in formal jobs compared to those who were housewives [23]. The authors also found that EBF practices were significantly lower among women who had a home delivery and who belonged to a family with a higher income [23].

Apart from the above-mentioned socioeconomic factors, other maternal and child characteristics including maternal nutritional status and frequency of antenatal check-ups amongst others have also been associated with early initiation of breastfeeding and EBF practices in Bangladesh [3, 5, 17]. However, the determinants of early initiation of breastfeeding and EBF practices may be differently distributed as per the socioeconomic status of the mothers. Investigating to what extent the socioeconomic inequalities in early initiation of breastfeeding and EBF exist, may help identify the underlying causes of these inequalities, and therefore inform the development of targeted breastfeeding interventions to mitigate these socioeconomic inequalities. Moreover, the socioeconomic inequalities in the key breastfeeding indicators (early initiation of breastfeeding and exclusive breastfeeding) may play an essential role in the overall prevalence of breastfeeding practices in Bangladesh. To the best of our knowledge, there are no studies in Bangladesh that examine the household wealth and mothers’ educational attainment inequalities in early initiation of breastfeeding and exclusive breastfeeding using socioeconomic analytical tools and based on the nationally representative sample of the 2018 Bangladesh Demographic and Health Survey (BDHS). Previous studies conducted in Bangladesh [24–28] and globally [29–31] also used women’s education, household wealth, or both, while investigating for socioeconomic inequalities, as these two indicators were identified as the most important measures of socioeconomic status, particularly in Bangladesh [24]. Therefore, the objective of this study was to examine the socioeconomic inequalities in early initiation of breastfeeding and EBF practices in Bangladesh based on the 2018 BDHS.

## Methods

### Data extraction

This study was a secondary data analysis of the 2018 BDHS [32]. For early initiation of breastfeeding, a total of 4950 women of reproductive age who had ever given birth were extracted. For exclusive breastfeeding, 924 children aged 0–5 months who were living with a respondent aged 15–49 years were extracted. BDHS is a vital source of data on early initiation of breastfeeding and EBF from a nationally representative sample of households.

### Sampling design

The nationally representative sample for the 2018 BDHS covers the entire population residing in non-institutional dwelling units in Bangladesh [32]. The survey used a list of enumeration areas (EAs) from the 2011 Population and Housing Census of the People’s Republic of Bangladesh, provided by the Bangladesh Bureau of Statistics (BBS). The primary sampling unit (PSU) of the survey is an EA with an average of about 120 households. Bangladesh consists of eight administrative divisions: Barishal, Chattogram, Dhaka, Khulna, Mymensingh, Rajshahi, Rangpur, and Sylhet. Each division is divided into zilas and each zila into upazilas. These divisions allow the country as a whole to be separated into rural and urban areas.

The survey was based on a two-stage stratified sample of households. In the first stage, 675 EAs (250 in urban areas and 425 in rural areas) were selected with probability proportional to EA size. The sample was drawn by BBS, following the specifications provided by the Inner City Fund (ICF) that include cluster allocation and instructions on sample selection. A complete household listing operation was then carried out in all the selected EAs to provide a sampling frame for the second-stage selection of households. In the second stage, a systematic sample of an average of 30 households per EA was selected to provide statistically reliable estimates of key demographic and health variables for the country as a whole, for urban and rural areas separately, and for each of the eight divisions. Based on this design, 20,250 residential households were selected. Completed interviews were expected from about 20,100 ever-married women aged 15–49 years [32].

### Selection and measurement of variables

#### Outcome variables

The primary outcomes of this study were early initiation of breastfeeding and exclusive breastfeeding. Early initiation of breastfeeding was determined from children who were put to the breast within the first hour of birth. Exclusive breastfeeding was estimated from children aged 0–5 months who were exclusively breastfed. Exclusive breastfeeding was determined based on the diets of infants during the 24-h before the survey (to avoid recall bias).

#### Socioeconomic variables

In this study, mother’s education and household wealth quintiles were used as measures of socioeconomic status. Therefore, the household wealth and mother’s education level inequalities in early initiation of breastfeeding and EBF were examined. Previous studies conducted in Bangladesh [24–28] and globally [29–31] also used women’s education, household wealth, or both, while investigating for socioeconomic inequalities, as these two indicators were identified as the most important measures of socioeconomic status, particularly in Bangladesh [24].

Mother’s education was categorised into groups (no formal education, primary, secondary, higher). The household wealth quintile was computed by DHS using the principal components analysis (PCA) technique. Scores were assigned and the wealth indicator variable was standardised using household assets such as floor type, wall type, roof type, water source, sanitation facilities, radio, electricity, television, refrigerator, cooking fuel, furniture, and number of persons per room. Then, the factor loadings and z-scores were calculated. For each household, the indicator values were multiplied by the loadings and summed to produce the household’s wealth index value. The standardised z-score was used to classify the overall scores to wealth quintiles (poorest, poorer, middle, richer, richest) [33].

#### Explanatory variables

The explanatory variables include: birth order (1st, 2nd, 3rd, 4th and above); sex of child (male, female); place of birth (home, health facility); antenatal care visit (< 4 visit, 4 + visit); mode of birth (vaginal, caesarean section); preceding birth interval (< 2 years, 2–4 years, 4 + years, first born); parity (1–2, 3–4, 5 +); age of mother (15–24 years, 25–34 years, 35 + years); residential status (urban, rural); age of mother at first birth (< 18 years, 18–24 years, 25 + years); marital status (married, not married); and geographical division (Barisal, Chittagong, Dhaka, Khulna, Mymensingh, Rajshahi, Rangpur, Sylhet).

The decision-making power was measured from a list of data elements (respondent involvement in decision on her healthcare, decision on large household purchases, and decision on visits to family or relatives). Mothers’ enlightenment level was measured using: educational attainment, read newspaper/magazines, listen to radio, and watch television. Using PCA, the factors were distilled into a more generalised set of weights that score “women’s enlightenment” and “decision making power” between 0 and 100. The standardised z-scores were used to disentangle the overall assigned scores to low, medium, and high [34, 35]. Wife beating was measured by aggregating responses from women and categorised into low and high. The following items were used: “beating justified if wife goes out without telling husband”, “beating justified if wife neglects the children”, “beating justified if wife argues with husband”, “beating justified if wife refuses to have sex with husband”, and “beating justified if wife burns the food”. Furthermore, neighbourhood socioeconomic disadvantage level was categorised into low, medium, and high using items such as rural residence, poorest household wealth status, no formal education, and not working [35].

### Statistical analysis

Stata version 14 (StataCorp., College Station, TX, USA) was used for data analysis. To account for the complex sampling design of BDHS, the survey module (‘svy’) command in Stata was used to adjust for stratification, clustering, sampling weights, and to calculate the weighted prevalence. Percentages were used in the univariate analysis. Lorenz curve and concentration index were used to examine the socioeconomic inequalities in early initiation of breastfeeding and exclusive breastfeeding [36, 37]. When the concentration index value was positive or the Lorenz curve lay below the diagonal line (line of equality), it indicated that early initiation of breastfeeding or EBF was greater among high socioeconomic groups (high household wealth and high educational attainment groups). Conversely, when the concentration index value was negative or the Lorenz curve lay above the diagonal line of equality, it showed that early initiation of breastfeeding or EBF was higher among low socioeconomic groups (low household wealth and low educational attainment groups). In the Lorenz curve, a higher degree of inequality was confirmed by how far away the curves sagged away from the line of equality. The explanatory variables were used for the stratified analyses. Concentration index were used to compute the contrast in early initiation of breastfeeding and exclusive breastfeeding [38]. The statistical significance was determined at *p* < 0.05.

## Results

### Socioeconomic inequalities in early initiation of breastfeeding

Table 1 illustrates the distribution of early initiation of breastfeeding among women of reproductive age in Bangladesh. The weighted prevalence of early initiation of breastfeeding was 60.8% (95% CI 59.0, 62.6%). Overall, a higher prevalence of early initiation of breastfeeding was observed among those with the following characteristics- female children, home births, < 4 antenatal care visits, vaginal delivery, mothers with 5 + children, rural residence, aged < 18 years at first birth, and from Sylhet geographical division. In addition, the prevalence of early initiation of breastfeeding varied with household wealth quintiles and educational attainment. The total estimates of early initiation of breastfeeding among poorest, poorer, middle, richer, and richest households were 67.8, 66.3, 58.4, 56.3, and 54.4%, respectively. Similarly, early initiation of breastfeeding prevalence of 64.4, 65.0, 61.1, and 52.3% were estimated for women with no formal education, primary, secondary, and higher education, respectively.

**Table 1:** Distribution of early initiation of breastfeeding among women of reproductive age in Bangladesh

Variable	n (%)	Early initiation of breastfeeding (%)	Early initiation of breastfeeding (%)								
			Household wealth quintile					Mother’s educational attainment			
			Poorest	Poorer	Middle	Richer	Richest	No formal education	Primary	Secondary	Higher
**Child characteristics/Mother’s pregnancy care**											
**Birth order**											
1st	1890 (38.2)	56.8	63.4	66.3	56.3	50.6	50.5	53.9	62.6	58.1	51.1
2nd	1620 (32.7)	60.7	66.0	65.0	58.8	58.3	55.8	62.3	65.4	61.0	51.4
3rd	847 (17.1)	64.8	72.3	69.8	57.0	60.4	59.7	69.1	64.9	64.3	62.9
4th and above	593 (12.0)	68.3	72.1	65.2	67.7	68.4	61.5	67.2	67.8	70.3	64.7
**Sex of child**											
Male	2596 (52.3)	60.6	67.7	67.3	57.7	54.9	54.5	62.0	65.8	61.0	51.5
Female	2354 (47.7)	61.0	67.9	65.3	59.3	57.7	54.3	66.9	64.2	61.2	53.3
**Place of birth**											
Home	2455 (49.6)	70.7	72.9	72.3	67.3	66.9	72.1	67.7	70.9	71.4	69.8
Health facility	2495 (50.4)	51.1	52.9	56.4	49.4	49.3	50.1	54.6	52.5	52.1	47.9
**Antenatal care**											
< 4 visit	2566 (51.8)	63.7	66.8	68.4	57.9	61.3	59.2	64.5	65.5	63.8	56.7
4+ visit	2384 (48.2)	57.7	70.0	63.0	59.1	52.1	52.5	63.9	64.2	58.6	50.6
**Mode of birth**											
Vaginal	3291 (66.6)	70.1	72.2	72.1	66.2	67.6	71.4	67.3	71.1	70.4	68.0
Caesarean section	1654 (33.4)	42.4	37.5	46.0	41.9	38.3	44.8	49.0	37.3	43.3	42.7
**Preceding birth interval**											
< 2 years	309 (6.2)	66.3	64.1	71.6	68.8	63.9	63.4	71.9	67.7	64.8	63.9
2–4 years	1330 (26.9)	63.0	70.5	65.1	61.5	60.2	53.9	65.1	64.2	65.3	50.9
4+ years	1406 (28.4)	63.3	70.6	67.4	57.5	60.1	59.7	66.4	67.6	61.3	56.3
First born	1905 (38.5)	56.5	63.0	67.4	56.0	50.7	50.5	53.9	62.1	57.8	51.2
**Parity**											
1–2	3510 (70.9)	58.6	64.7	65.7	57.4	53.9	52.9	58.4	64.1	59.5	51.2
3–4	1199 (24.2)	65.6	71.1	68.5	61.8	62.1	59.8	66.1	65.4	66.0	62.8
5+	241 (4.9)	69.7	75.7	64.9	58.1	71.1	66.7	70.7	69.8	67.4	100.0
**Mothers sociodemographic characteristics**											
**Age of mother**											
15–24	2615 (52.8)	59.5	65.5	66.4	58.5	53.5	51.8	59.4	64.4	60.6	50.1
25–34	2030 (41.0)	62.3	69.4	66.7	59.3	60.4	56.0	66.0	66.6	61.6	55.4
35+	305 (6.2)	62.0	76.2	64.1	52.0	54.9	59.7	68.2	60.7	64.5	52.9
**Residential status**											
Urban	1700 (34.3)	59.4	71.4	67.1	59.8	56.8	56.1	65.0	63.6	60.6	52.0
Rural	3250 (65.7)	61.6	67.0	66.2	57.9	56.0	50.7	64.1	65.7	61.3	52.7
**Age at first birth**											
< 18	2007 (40.5)	63.6	69.7	67.8	59.5	56.6	57.5	63.9	67.4	62.6	48.0
18–24	2663 (53.8)	52.7	65.7	64.5	58.5	56.7	54.5	66.2	62.6	60.6	53.2
25+	280 (5.7)	52.1	58.8	73.1	48.8	50.9	48.9	50.0	61.8	48.5	51.8
**Marital status**											
Married	4886 (98.7)	60.8	67.8	66.3	58.6	56.2	54.6	64.1	65.2	61.1	52.5
Not married	64 (1.3)	59.4	66.7	68.4	50.0	71.4	41.7	75.0	55.0	63.3	33.3
**Geographical division**											
Barisal	528 (10.7)	63.1	63.5	69.4	59.4	58.3	62.0	64.3	66.7	64.7	53.5
Chittagong	824 (16.7)	55.0	74.7	56.4	52.1	52.8	45.5	59.2	60.3	54.8	47.3
Dhaka	728 (14.7)	63.1	73.3	65.9	59.7	57.9	64.9	57.1	66.0	64.2	58.8
Khulna	517 (10.4)	50.7	56.3	68.8	41.5	45.8	44.3	26.7	60.4	50.8	43.9
Mymensingh	599 (12.1)	64.1	67.7	64.4	68.9	57.0	55.9	62.5	63.1	67.8	57.1
Rajshahi	520 (10.5)	59.6	56.5	62.7	62.5	56.5	58.7	57.1	66.9	58.8	52.6
Rangpur	553 (11.2)	64.9	70.2	69.7	61.2	61.5	48.6	74.2	67.7	68.3	52.1
Sylhet	681 (13.8)	66.1	71.9	72.9	70.0	63.9	52.7	80.9	67.7	64.9	52.4
**Mothers empowerment**											
**Decision making power**											
Low	1930 (39.5)	58.8	66.6	66.0	55.1	52.9	53.2	57.6	63.3	59.4	50.8
High	2956 (60.5)	62.1	68.4	66.5	61.1	58.6	55.3	67.3	66.3	62.3	53.5
**Mother’s enlightenment**											
Low	1885 (38.1)	66.5	69.0	69.5	61.6	59.1	62.1	64.6	68.4	67.1	57.7
Medium	2354 (47.6)	58.7	64.0	63.9	58.7	55.8	55.7	0.0	61.8	58.7	53.5
High	711 (14.4)	52.7	61.9	61.5	46.8	54.8	50.3	–	60.5	56.2	49.4
**Wife beating**											
Low	4068 (82.2)	60.6	68.0	65.6	58.4	56.5	54.7	63.0	64.9	61.4	52.2
High	882 (17.8)	61.9	66.9	69.2	58.7	55.4	51.8	68.9	65.4	59.7	54.2
**Neighbourhood socioeconomic disadvantage**											
Low	1656 (33.5)	56.2	68.9	61.4	56.5	56.1	54.3	60.8	61.2	56.8	50.6
Medium	1649 (33.3)	59.8	62.4	65.6	57.6	55.6	55.7	56.9	60.8	61.1	55.6
High	1645 (33.2)	66.5	69.4	68.8	61.7	58.7	51.0	68.3	70.4	66.0	51.0
**Total estimates of early initiation of breastfeeding**	**4950**	**60.8**	**67.8**	**66.3**	**58.4**	**56.3**	**54.4**	**64.4**	**65.0**	**61.1**	**52.3**

**Fig. 1:**
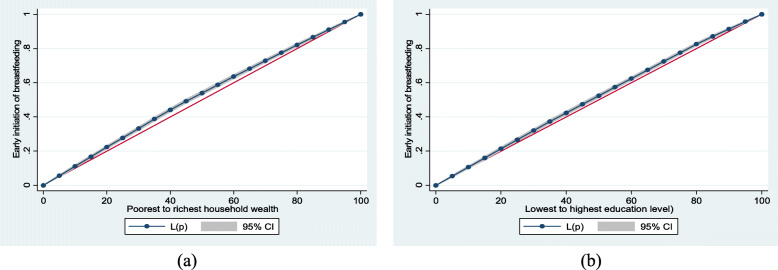
Early initiation of breastfeeding by (**a**) household wealth (**b**) education level

Household wealth and mother’s education level inequalities in early initiation of breastfeeding in Bangladesh were examined using the Lorenz curve. Early initiation of breastfeeding was higher among lower household wealth groups (Fig. 1a) and among mothers with lower education levels (Fig. 1b) as the Lorenz curves lay above the lines of equality.

Household wealth and mothers’ educational attainment inequalities in early initiation of breastfeeding across selected child and mother’s characteristics are outlined in Table 2. Early initiation of breastfeeding was higher in lower household wealth groups particularly among 1st (Conc. Index = − 0.059; SE = 0.011), 2nd (Conc. Index = − 0.036; SE = 0.011) and 3rd (Conc. Index = − 0.046; SE = 0.014) order births; male (Conc. Index = − 0.052; SE = 0.009) and female (Conc. Index = − 0.046; SE = 0.009); home delivery (Conc. Index = − 0.015; SE = 0.007); < 4 antenatal care visits (Conc. Index = − 0.028; SE = 0.008) and 4+ antenatal care visits (Conc. Index = − 0.057; SE = 0.010). However, there were significant differences in the indices for antenatal care visits (*p* = 0.023) and geographical division (*p* = 0.002) in household wealth inequalities in early initiation of breastfeeding. In addition, early initiation of breastfeeding was higher among mothers with lower educational attainment particularly among 1st (Conc. Index = − 0.035; SE = 0.011) and 2nd (Conc. Index = − 0.034; SE = 0.010) order births; male (Conc. Index = − 0.039; SE = 0.008) and female (Conc. Index = − 0.032; SE = 0.009); and 4+ antenatal care visits (Conc. Index = − 0.044; SE = 0.009). Conversely, there were significant differences in the indices for antenatal care visits (*p* = 0.020) and parity (*p* = 0.033) in mother’s education inequalities in early initiation of breastfeeding. Overall, early initiation of breastfeeding was higher among lower household wealth groups (Conc. Index = − 0.049; SE = 0.006) and lower educational attainment groups (Conc. Index = − 0.035; SE = 0.006) among women in Bangladesh.

**Table 2:** Household wealth and educational attainment inequalities in early initiation of breastfeeding

Variable	Household wealth quintile		Mother’s education	
	Concentration index (SE)	P^@^	Concentration index (SE)	P^@^
**Child characteristics**				
**Birth order**		0.266		0.088
1st	− 0.059 (0.011)*		−0.035 (0.011)*	
2nd	−0.036 (0.011)*		−0.034 (0.010)*	
3rd	−0.046 (0.014)*		−0.009 (0.013)	
4th and above	−0.021 (0.016)		0.007 (0.015)	
**Sex of child**		0.663		0.655
Male	−0.052 (0.009)*		−0.039 (0.008)*	
Female	−0.046 (0.009)*		−0.032 (0.009)*	
**Place of birth**		0.875		0.059
Home	− 0.015 (0.007)*		0.004 (0.007)	
Health facility	−0.017 (0.011)		−0.019 (0.010)	
**Antenatal care**		0.023*		0.020*
< 4 visit	−0.028 (0.008)*		−0.016 (0.008)	
4+ visit	−0.057 (0.010)*		−0.044 (0.009)*	
**Mode of birth**		0.066		0.431
Vaginal	−0.012 (0.006)		−0.002 (0.006)	
Caesarean section	0.015 (0.016)		0.009 (0.015)	
**Preceding birth interval**		0.236		0.874
< 2 years	−0.004 (0.023)		−0.018 (0.022)	
2–4 years	−0.051 (0.012)*		−0.023 (0.011)*	
4+ years	−0.038 (0.011)*		−0.029 (0.011)*	
First born	−0.056 (0.011)*		−0.033 (0.011)*	
**Parity**		0.694		0.033*
1–2	−0.048 (0.008)*		−0.037 (0.007)*	
3–4	−0.037 (0.012)*		−0.002 (0.011)	
5+	−0.032 (0.023)		−0.006 (0.023)	
**Mothers sociodemographic characteristics**				
**Age of mother**		0.759		0.927
15–24	−0.054 (0.009)*		−0.037 (0.008)*	
25–34	−0.044 (0.010)*		−0.032 (0.009)*	
35+	−0.053 (0.025)*		−0.032 (0.025)	
**Residential status**		0.492		0.531
Urban	−0.040 (0.011)*		−0.040 (0.011)*	
Rural	−0.050 (0.008)*		−0.032 (0.007)*	
**Age at first birth**		0.895		0.670
< 18	−0.047 (0.009)*		−0.025 (0.009)*	
18–24	−0.041 (0.009)*		−0.032 (0.009)*	
25+	−0.046 (0.031)		−0.011 (0.029)	
**Marital status**		0.658		0.852
Married	−0.049 (0.006)*		−0.035 (0.006)*	
Not married	−0.074 (0.058)		−0.045 (0.056)	
**Geographical division**		0.002*		0.485
Barisal	−0.017 (0.019)		−0.033 (0.018)	
Chittagong	−0.087 (0.017)*		−0.040 (0.016)*	
Dhaka	−0.005 (0.016)		−0.011 (0.015)	
Khulna	−0.077 (0.024)*		−0.035 (0.022)	
Mymensingh	−0.030 (0.017)		−0.004 (0.017)	
Rajshahi	−0.005 (0.020)		−0.039 (0.019)*	
Rangpur	−0.054 (0.017)*		−0.046 (0.017)*	
Sylhet	−0.060 (0.015)*		−0.048 (0.015)*	
**Mothers empowerment**				
**Decision making power**		0.486		0.565
Low	−0.054 (0.011)*		−0.030 (0.010)*	
High	−0.045 (0.008)*		−0.038 (0.008)*	
**Mother’s enlightenment**		0.956		0.212
Low	−0.027 (0.009)*		−0.006 ()0.009	
Medium	−0.031 (0.019)*		−0.022 (0.009)*	
High	−0.029 (0.019)		−0.037 (0.018)*	
**Wife beating**		0.772		0.943
Low	−0.048 (0.007)*		−0.035 (0.007)*	
High	−0.053 (0.015)*		−0.034 (0.014)*	
**Neighbourhood socioeconomic disadvantage**		0.712		0.183
Low	−0.022 (0.012)		−0.036 (0.011)*	
Medium	−0.033 (0.011)*		−0.010 (0.011)	
High	−0.032 (0.009)*		−0.032 (0.009)*	
**Total estimates**	−0.049 (0.006)*		−0.035 (0.006)*	

*Significant at p < 0.05

*SE* Standard error

**P**^**@**^ = *p*-value comparing indices across the levels of a variable

### Socioeconomic inequalities in exclusive breastfeeding

Table 3 shows the distribution of EBF among children aged 0–5 months in Bangladesh. The weighted prevalence of EBF was 66.8% (95% CI 63.1, 70.3%). Overall, a higher prevalence of EBF was observed among those with the following characteristics- female children, health facility-based delivery, 4+ antenatal care visits, vaginal delivery, mothers with 1–2 children, rural residence, aged 18–24 years at first birth, and from Chittagong geographical region. The total estimates of EBF among poorest, poorer, middle, richer, and richest households were 63.0, 65.2, 67.7, 66.7, and 69.9%, respectively. Similarly, 62.5, 66.0, 66.3, and 68.9% were estimated for women with no formal education, primary, secondary and higher education, respectively.

**Table 3:** Distribution of exclusive breastfeeding among children under 6 months in Bangladesh

Variable	n (%)	Exclusive breastfeeding (%)	Exclusive breastfeeding (%)								
			Household wealth quintile					Mother’s educational attainment			
			Poorest	Poorer	Middle	Richer	Richest	No formal education	Primary	Secondary	Higher
**Child characteristics**											
**Birth order**											
1st	339 (36.7)	68.1	69.0	59.4	67.1	67.6	77.1	69.2	65.8	68.3	69.4
2nd	296 (32.0)	66.2	58.5	67.4	70.3	74.0	63.2	68.8	66.7	64.7	69.1
3rd	171 (18.5)	63.2	63.9	63.9	66.7	46.2	70.0	60.0	62.9	64.3	60.0
4th and above	118 (12.8)	67.0	61.0	75.0	62.5	71.4	62.5	56.0	69.1	69.4	100.0
**Sex of child**											
Male	480 (52.0)	65.4	62.5	58.8	66.0	68.2	70.6	55.6	66.9	65.6	65.7
Female	444 (48.1)	67.6	63.6	70.3	69.6	65.1	68.8	67.6	65.2	67.0	73.6
**Place of birth**											
Home	453 (49.0)	65.1	61.6	66.4	71.0	67.9	55.6	63.8	65.5	65.3	64.3
Health facility	471 (51.0)	67.7	66.7	62.7	64.5	66.1	73.3	58.8	67.0	67.1	70.3
**Antenatal care**											
< 4 visit	502 (54.8)	66.5	65.2	63.3	70.5	61.5	74.6	66.0	65.3	68.4	64.1
4+ visit	414 (45.2)	67.6	60.3	68.8	64.2	71.7	70.2	50.0	68.2	65.0	73.5
**Mode of birth**											
Vaginal	605 (65.5)	66.6	62.9	65.5	69.2	70.5	68.7	66.7	64.2	68.1	68.2
Caesarean section	318 (34.5)	66.0	64.0	63.6	65.2	61.8	70.3	46.2	74.0	62.7	69.0
**Preceding birth interval**											
< 2 years	56 (6.1)	64.3	43.8	66.7	46.2	100.0	85.7	28.6	81.3	58.6	100.0
2–4 years	241 (26.1)	71.8	74.2	79.0	71.4	60.6	66.7	79.0	73.6	70.0	68.6
4+ years	285 (30.8)	61.1	52.4	54.4	70.7	62.3	64.6	56.0	56.8	63.6	65.6
First born	342 (37.0)	67.5	67.8	59.4	67.1	67.6	75.0	69.2	64.9	67.8	68.8
**Parity**											
1–2	632 (68.4)	67.6	63.4	62.8	68.6	70.2	71.9	71.4	66.2	66.4	70.2
3–4	250 (27.1)	63.6	64.4	66.1	64.4	55.0	66.0	59.3	63.8	65.5	57.9
5+	42 (4.6)	66.7	55.6	83.3	75.0	71.4	0.0	44.4	73.9	70.0	–
**Mothers sociodemographic characteristics**											
**Age of mother**											
15–24	535 (57.9)	68.0	67.2	65.8	65.8	71.6	70.8	67.7	66.2	68.5	69.4
25–34	347 (37.6)	66.0	59.7	66.7	71.4	60.3	70.7	65.2	68.5	62.9	69.2
35+	42 (4.5)	50.0	33.3	50.0	50.0	50.0	60.0	28.6	46.7	61.5	57.1
**Residential status**											
Urban	328 (35.5)	62.5	59.5	55.9	59.6	59.5	68.3	51.9	64.2	61.0	66.3
Rural	596 (64.5)	68.6	63.9	67.4	70.9	72.2	73.3	70.3	67.1	68.5	71.1
**Age at first birth**											
< 18	351 (38.0)	63.3	60.8	64.5	60.8	66.1	66.7	62.5	66.9	61.4	56.3
18–24	510 (55.2)	68.6	65.7	66.7	73.0	67.3	70.2	63.3	64.8	69.9	71.5
25+	63 (6.8)	66.7	50.0	55.6	66.7	62.5	73.3	50.0	70.0	70.0	65.9
**Marital status**											
Married	918 (99.3)	66.5	62.8	65.6	68.1	66.5	69.6	62.5	66.2	66.3	68.7
Not married	6 (0.7)	66.7	100.0	0.0	0.0	100.0	100.0	–	50.0	66.7	100.0
**Geographical division**											
Barisal	106 (11.5)	65.1	71.4	48.0	58.3	78.6	87.5	85.7	65.6	56.8	73.9
Chittagong	162 (17.5)	79.6	81.5	90.0	85.0	71.4	74.5	66.7	88.3	76.5	80.8
Dhaka	128 (13.9)	53.9	33.3	46.2	60.0	68.3	46.2	50.0	55.0	53.5	55.0
Khulna	98 (10.6)	57.1	44.4	64.7	54.2	65.2	52.0	50.0	55.0	63.2	41.2
Mymensingh	113 (12.2)	54.0	41.9	48.5	76.2	35.3	90,9	63.6	53.1	46.8	65.2
Rajshahi	85 (9.2)	72.9	66.7	78.3	60.0	76.2	83.3	100.0	62.5	76.7	72.0
Rangpur	102 (11.0)	72.6	66.7	70.0	84.6	55.6	93.3	60.0	61.3	75.6	84.0
Sylhet	130 (14.1)	72.3	72.4	76.7	58.3	72.2	79.3	57.1	72.6	79.6	66.7
**Mothers empowerment**											
**Decision making power**											
Low	398 (43.4)	67.3	62.2	66.3	69.0	72.9	65.2	76.0	66.1	67.5	65.7
High	520 (56.6)	65.8	63.2	65.0	67.4	60.0	72.2	53.9	66.2	65.1	70.6
**Mother’s enlightenment**											
Low	390 (42.2)	66.7	63.9	68.6	70.3	68.2	64.7	62.5	65.7	67.6	74.3
Medium	399 (43.2)	66.2	60.0	66.2	67.0	65.0	67.9	–	67.0	65.4	67.1
High	135 (14.6)	66.7	50.0	47.6	61.1	70.0	75.0	–	57.1	66.0	68.0
**Wife beating**											
Low	758 (82.0)	66.8	63.2	62.8	68.9	68.0	70.6	60.8	67.5	66.9	67.5
High	166 (18.0)	65.1	62.5	75.8	62.9	58.3	65.4	69.2	61.5	63.6	90.9
**Neighbourhood socioeconomic disadvantage**											
Low	306 (33.1)	68.6	80.0	68.0	67.8	67.9	69.1	66.7	69.4	68.4	68.7
Medium	318 (34.4)	64.2	53.1	63.6	65.9	63.8	75.6	35.7	65.9	63.5	68.9
High	300 (32.5)	66.7	65.8	66.2	71.1	68.8	55.6	71.1	63.9	67.2	69.6
**Total estimates of exclusive breastfeeding**	**924**	**66.8**	**63.0**	**65.2**	**67.7**	**66.7**	**69.9**	**62.5**	**66.0**	**66.3**	**68.9**

**Fig. 2:**
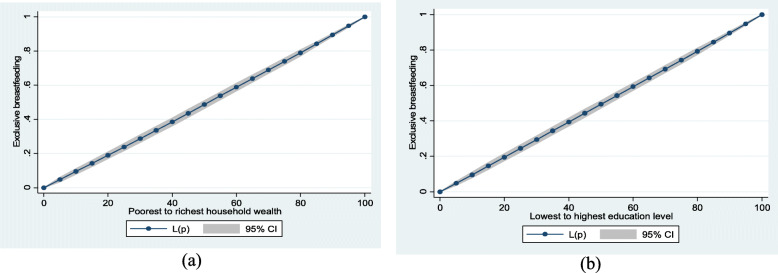
Exclusive breastfeeding by (**a**) household wealth (**b**) education level

Household wealth and mother’s education level inequalities in EBF in Bangladesh were examined using the Lorenz curve. Figs. 2a and Fig. 2b showed no significant household wealth and mother’s education level inequalities in EBF, respectively.

Household wealth and mothers’ educational attainment inequalities in EBF across selected child and mother’s characteristics are presented in Table 4. Exclusive breastfeeding was higher in higher household wealth groups particularly among < 2 years preceding birth interval (Conc. Index = 0.152; SE = 0.053); Mymensingh geographical division (Conc. Index = 0.100; SE = 0.048); and mothers with high enlightenment (Conc. Index = 0.079; SE = 0.032). However, there were significant differences in the indices for preceding birth interval (*p* = 0.011) in household wealth inequalities in exclusive breastfeeding. In addition, EBF was higher in higher among mothers with higher educational attainment in Rangpur geographical division (Conc. Index = 0.067; SE = 0.033).

**Table 4:** Household wealth and educational attainment inequalities in exclusive breastfeeding

Variable	Household wealth quintile		Mother’s education	
	Concentration index (SE)	P^@^	Concentration index (SE)	P^@^
**Child characteristics**				
**Birth order**		0.892		0.802
1st	0.031 (0.021)		0.009 (0.020)	
2nd	0.018 (0.024)		0.002 (0.022)	
3rd	0.001 (0.033)		0.002 (0.031)	
4th and above	0.020 (0.036)		0.041 (0.035)	
**Sex of child**		0.336		0.640
Male	0.031 (0.019)		0.005 (0.018)	
Female	0.006 (0.019)		0.017 (0.018)	
**Place of birth**		0.529		0.581
Home	0.011 (0.019)		0.001 (0.018)	
Health facility	0.028 (0.018)		0.014 (0.017)	
**Antenatal care**		0.747		0.440
< 4 visit	0.017 (0.018)		0.003 (0.017)	
4+ visit	0.025 (0.019)		0.023 (0.018)	
**Mode of birth**		0.944		0.986
Vaginal	0.023 (0.016)		0.012 (0.015)	
Caesarean section	0.021 (0.022)		0.011 (0.022)	
**Preceding birth interval**		0.011*		0.419
< 2 years	0.152 (0.053)*		0.047 (0.053)	
2–4 years	−0.034 (0.023)		−0.020 (0.022)	
4+ years	0.042 (0.027)		0.031 (0.025)	
First born	0.029 (0.021)		0.009 (0.020)	
**Parity**		0.442		0.657
1–2	0.028 (0.016)		0.008 (0.015)	
3–4	−0.008 (0.027)		0.005 (0.026)	
5+	0.043 (0.061)		0.064 (0.057)	
**Mothers sociodemographic characteristics**				
**Age of mother**		0.409		0.182
15–24	0.014 (0.017)		0.008 (0.016)	
25–34	0.022 (0.022)		−0.002 (0.021)	
35+	0.102 (0.086)		0.112 (0.086)	
**Residential status**		0.750		0.677
Urban	0.037 (0.024)		0.019 (0.023)	
Rural	0.028 (0.016)		0.007 (0.015)	
**Age at first birth**		0.702		0.0293
< 18	0.015 (0.023)		−0.020 (0.021)	
18–24	0.011 (0.017)		0.021 (0.016)	
25+	0.058 (0.048)		−0.005 (0.044)	
**Marital status**		0.410		0.476
Married	0.018 (0.013)		0.010 (0.013)	
Not married	0.167 (0.180)		0.125 (0.176)	
**Geographical division**		0.349		0.835
Barisal	0.020 (0.040)		−0.006 (0.039)	
Chittagong	−0.033 (0.022)		−0.007 (0.021)	
Dhaka	0.018 (0.046)		0.004 (0.044)	
Khulna	−0.002 (0.050)		−0.025 (0.045)	
Mymensingh	0.100 (0.048)*		0.011 (0.048)	
Rajshahi	0.021 (0.038)		0.012 (0.035)	
Rangpur	0.051 (0.033)		0.067 (0.033)*	
Sylhet	0.009 (0.031)		0.021 (0.030)	
**Mothers empowerment**				
**Decision making power**		0.915		0.232
Low	0.016 (0.020)		−0.008 (0.019)	
High	0.019 (0.018)		0.023 (0.017)	
**Mother’s enlightenment**		0.193		0.692
Low	0.017 (0.020)		0.022 (0.020)	
Medium	0.009 (0.020)		−0.001 (0.019)	
High	0.079 (0.032)*		0.014 (0.031)	
**Wife beating**		0.351		0.556
Low	0.024 (0.014)		0.006 (0.014)	
High	−0.008 (0.032)		0.025 (0.030)	
**Neighbourhood socioeconomic disadvantage**		0.332		0.621
Low	0.002 (0.021)		−0.001 (0.021)	
Medium	0.044 (0.024)		0.027 (0.023)	
High	0.007 (0.022)		0.003 (0.022)	
**Total estimates**	0.019 (0.013)		0.010 (0.013)	

*Significant at p < 0.05

*SE* Standard error

**P**^**@**^ = *p*-value comparing indices across the levels of a variable

## Discussion

To the best of our knowledge, this study is one of the first to examine the socioeconomic inequalities in key breastfeeding indicators (early initiation of breastfeeding and exclusive breastfeeding) in Bangladesh based on a nationally representative sample from the 2018 BDHS. Despite the known benefits of optimal breastfeeding practices, the present study demonstrated that only about three in five infants received breast milk within the first hour of birth, while only about two-thirds of children aged 0–5 months were exclusively breastfed. In this study, significant socioeconomic inequalities in early initiation of breastfeeding were observed. Interestingly, the prevalence of early initiation of breastfeeding was higher among those with lower household wealth and lower educational attainment. Conversely, although significant socioeconomic inequalities in EBF were not found, EBF was higher among higher household wealth and higher educational attainment groups across a few child and mother’s characteristics.

In the current study, the prevalence of early initiation of breastfeeding and EBF among women in Bangladesh were only 60.8 and 66.8%, respectively. The study findings indicate that the breastfeeding recommendations are not being met among childbearing women in Bangladesh. The prevalence of early initiation of breastfeeding was lower than the 66.7% [39], and the prevalence of EBF was slightly higher the 65.0 and 64.0% [17, 18] reported in previous studies conducted in Bangladesh. In comparison, both early initiation of breastfeeding and exclusive breastfeeding rates were higher than in LMICs, where only around 50% of infants receive breast milk within the first hour of birth [40], while only 37% of infants under six months of age are exclusively breastfed [41]. The early initiation of breastfeeding and exclusive breastfeeding prevalence rates were also comparable with the results presented by a study that has compiled the breastfeeding rates of various South Asian countries [42]. Other studies conducted in various LMICs [13–16] have reported similar or higher early initiation of breastfeeding and EBF rates yet have constantly emphasised the need to increase breastfeeding rates to ensure better health outcomes.

Comparatively, a higher prevalence of early initiation of breastfeeding and EBF were observed among those who had a vaginal delivery and lived in a rural residence. Caesarean section has been identified as a barrier to EBF in Pakistan, and a barrier to early initiation of breastfeeding in most South Asian countries including India, Nepal and Pakistan [42]. A study conducted in Sri Lanka also found lower EBF rates among mothers residing in urban areas [22]. In this study, although early initiation of breastfeeding prevalence was higher among home births, exclusive breastfeeding prevalence was higher among health-facility based delivery. Conversely, higher rates of early initiation of breastfeeding and EBF were observed among health-facility delivery and home delivery, respectively, as reported by breastfeeding studies in Nepal [43, 44]. Nonetheless, previous studies conducted in other developed and developing countries have reported similar findings indicating a significant association of the aforementioned factors with early initiation of breastfeeding and exclusive breastfeeding [13, 16, 45–47].

In this study, the overall low prevalence of early initiation of breastfeeding and EBF may be a result of various factors including low support from family members, lack of formal education, limited awareness and knowledge about breastfeeding practices, lack of advice from health staff during antenatal visits, and deliveries in non-baby friendly institutions, as indicated by prior breastfeeding studies [13, 45]. To increase breastfeeding rates, the Baby-Friendly Hospital Initiative was established by the WHO and UNICEF as a hospital-based intervention, where breastfeeding is supported, practiced, protected, and promoted [48]. However, with only 39.7% of hospital-based deliveries in Bangladesh [49], underutilisation of hospital-based interventions is unavoidable. Apart from poverty, the most common reasons for preferring home delivery with a traditional birth attendant in Bangladesh include traditional views, religious misconception, poor road conditions, limited decision making power of women in the family, and lack of transportation to reach the nearest health facility [50]. Besides, many people in Bangladesh prefer home delivery due to the lack of knowledge and awareness about service delivery points, fear of increased chances of having a caesarean delivery at the hospital, and a lack of female doctors in healthcare facilities [50].

In the present study, household wealth and mothers’ educational attainment inequalities in early initiation of breastfeeding were observed. Previous studies conducted in both developed and developing countries have consistently found a significant association of mothers’ education and socioeconomic status with early initiation of breastfeeding [13, 16, 45, 46]. In this study, a deep dive analysis showed that wealth-related and mother’s education inequalities in early initiation of breastfeeding varied with the selected child and maternal characteristics. Overall, early initiation of breastfeeding prevalence was higher among lower household wealth and lower maternal education level groups. On the contrary, several prior studies have reported that mothers who attended formal education or had higher household wealth were associated with increased odds of early initiation of breastfeeding [51–54]. In South Asian studies, early initiation of breastfeeding prevalence was found to be lower among those from richer wealth quintile in Sri Lanka similar to the findings of this study [22]; however, those with lower educational status have been found to have lower prevalence rates in Nepal [55]. A lack of awareness, unavailability, or inaccessibility to breastfeeding alternatives such as infant formula feeding among mothers with lower educational level and lower household wealth, are some factors which may increase the likelihood of breastfeeding as compared to bottle feeding in the first hour of birth [15, 53]. In LMICs, breastfeeding is one of the few positive health behaviours with a higher prevalence in lower socioeconomic groups [41]. These findings suggest that breastfeeding is contributing to reducing health gaps between children from high and low socioeconomic groups to some extent.

The higher early initiation of breastfeeding prevalence among lower socioeconomic groups in this study may also be linked with the concentrated effort of maternal and child health intervention programs in Bangladesh [56]. However, this was not the case with exclusive breastfeeding practices as EBF prevalence was higher among higher household wealth and higher educational attainment groups across a few child and mother’s characteristics, similar to the findings of a prior study [57]. Research suggests that mothers with higher education and household wealth are more knowledgeable in the appropriate utilization of maternal healthcare services including antenatal care (ANC), postnatal care (PNC), and institutional delivery, which are essential measures to scale up EBF practices [58–61]. Underutilization of both ANC and PNC services have also been observed among Nepalese mothers with a lower household wealth and educational status [62, 63]. In developing countries, women with a higher socioeconomic status may also have better access to health promotion and infant feeding messages and may better respond to them as compared to those with lower socioeconomic status [64]. Similarly, low maternal education has been identified as a major barrier of EBF in some LMICs [65, 66], which suggests that maternal education plays an essential role in infant nutrition and development. Primary education has been identified as the basic threshold to benefit from health information, therefore, at least acquiring a primary education for those with no formal education may empower women from marginalized groups to act on health information, particularly those targeted at promoting exclusive breastfeeding [64]. On the other hand, significant barriers to EBF have been identified in literature which could be applicable for all mothers irrespective of their socioeconomic status, including the long-term commitment required from mothers’ in order to continue to exclusively breastfeed their children until six months of age, and the difficulties associated with continuing to breastfeed while attending formal or informal employment [17]. Other factors may include, but are not limited to, a lack of planning for exclusive breastfeeding during pregnancy, prelacteal feeding due to some cultural norms, and delivery by a caesarean section [67].

It may be difficult to improve EBF practices among poor women who lack formal education, particularly when the majority of births take place in homes and are assisted by skilled/traditional birth attendants (TBA) in Bangladesh [50]. Nonetheless, social and behavioral change interventions [68], such as maternal counselling [69], mobile phone follow-up services [70], and training TBAs [71] to improve breastfeeding practices for home births in Bangladesh may be effective. Furthermore, a recent study reported a number of interventions such as education and counselling, maternal and newborn health initiatives, community mobilization, and mass media, delivered across several implementation environments- health facility, community, and home/family, were effective in improving breastfeeding practices including early initiation of breastfeeding and EBF in various South Asian countries [72].In LMICs, wealthier and educated mothers are also more likely to deliver through a caesarean section as compared to vaginal delivery [73, 74]. Caesarean section has been identified as a barrier to early initiation of breastfeeding due to several key reasons that delay breastfeeding initiation including longer hospital stay, delayed skin-to-skin contact, and prolonged mother-infant separation [75]. Moreover, almost all caesarean section takes place in health facilities, which accounts for over 60% of the institutional deliveries in Bangladesh [76]. High rates of caesarean section in Bangladesh have been associated with previous maternal experience in caesarean section, high risk pregnancies, complications during labour, and maternal or physician preferences [77, 78]. This highlights the need to address early initiation of breastfeeding practices in a post-caesarean section setting, and design interventions that facilitate early initiation of breastfeeding among women who had a health facility-based delivery [20]. It is also important to improve health services facilities and build capacity among the healthcare providers [20]. A recent study carried out in Bangladesh also reported that early initiation of breastfeeding practices were higher in district hospitals; in hospitals having visual privacy in the delivery room; if mothers immediately examined by healthcare providers; and if babies had skin-to-skin contact with mothers immediately after delivery [79]. Further strategies to promote early initiation of breastfeeding in a health facility may include staff education, training, and participation, and strong leadership and development of a strategic approach [20].

The overall lower prevalence of early initiation of breastfeeding and EBF in Bangladesh suggest that an improvement in breastfeeding practices is required to achieve the Sustainable Development Goals [80]. The promotion of breastfeeding is a key component of child growth mechanisms irrespective of the status of children and mothers. The study findings indicate that Bangladesh is yet to fully institutionalise the goals of the International Code of Marketing of Breast-milk Substitutes [81], Global Strategy for Infant and Young Child Feeding [4], and Baby-Friendly Hospital Initiative [82] in the uptake of early initiation of breastfeeding and exclusive breastfeeding.

### Strengths and limitations

The present study has several strengths. Firstly, this study used high-quality and nationally representative data covering a large sample size from the 2018 BDHS [32], which enables the results to be generalisable to women in Bangladesh. Secondly, appropriate statistical adjustments were performed for the survey designs to ensure that the results of this study were more reliable. Finally, with the use of vital socioeconomic tools, this study is one of the first to examine the socioeconomic inequalities in early initiation of breastfeeding and EBF practices in Bangladesh.

Nonetheless, this study has a few limitations. Firstly, there might be a potential for recall bias which could lead to underestimation or overestimation of the outcome variables. Mothers were asked how their child was fed in the preceding day and the capacity to recall food might vary by key factors examined in the analysis such as maternal educational level. Further, the definition of EBF was based on 24-h recall, and the day-to-day variability in food intake might lead to recall or measurement bias. However, similar to prior studies [14, 15], recall bias was minimised by restricting the analysis to children under six months of age who were living with the mother. Secondly, DHS does not collect data on household income or expenditure, which are the traditional indicators used to measure wealth status. The assets-based wealth index was used as a proxy indicator for household economic status, and it does not always produce the exact results when compared with those obtained from direct measurements of income and expenditure where such data are available or can be collected reliably.

## Conclusions

This study examined the socioeconomic inequalities in key breastfeeding indicators, namely early initiation of breastfeeding and exclusive breastfeeding in Bangladesh. Improving optimal breastfeeding practices in Bangladesh should be given utmost priority to meet the global breastfeeding targets. A need to address the socioeconomic inequalities in breastfeeding practices was also identified. Appropriate health promotion programs individually targeted at mothers from both high and low socioeconomic groups are critical to ensure optimal breastfeeding practices in Bangladesh. Furthermore, outreach programs to ensure healthcare service utilisation throughout pregnancy and delivery and to increase knowledge and awareness of breastfeeding practices are vital measures to enhance uptake and practice of early initiation of breastfeeding and exclusive breastfeeding in Bangladesh.

## Data Availability

Data for this study were sourced from the Demographic and Health surveys (DHS) and are available online at: http://dhsprogram.com/data/available-datasets.cfm.

## Abbreviations

BBS: Bangladesh Bureau of Statistics
BDHS: Bangladesh Demographic and Health Survey
CI: Confidence Interval
Conc: Concentration
EAs: Enumeration Areas
EBF: Exclusive Breastfeeding
ICF: Inner City Fund
LMICs: Low- and middle-income countries
PCA: Principal Components Analysis
PSU: Primary Sampling Unit
SE: Standard Error
TX: Texas
UNICEF: United Nations International Children’s Emergency Fund
USA: United States of America
WHO: World Health Organization
